# An Immunohistochemical Study on Ki-67 Expression in Squamous Cell Carcinomas of Cervix With Clinicopathological Correlation

**DOI:** 10.7759/cureus.34155

**Published:** 2023-01-24

**Authors:** Devaki Priyanka R, Sundaram Arunachalam, Kalaivani Amitkumar, Jaison Jacob John, Muthu Sudalaimuthu

**Affiliations:** 1 Department of Pathology, SRM Medical College Hospital and Research Centre, Chennai, IND

**Keywords:** keratinizing squamous cell carcinoma, clinical stage, histological grade, ki-67, squamous cell carcinoma, cervical carcinoma

## Abstract

Background

Cervical carcinoma is one of the most prevalent cancers affecting women worldwide. Studies on Ki-67 expression in cervical lesions had focused mainly on the intraepithelial lesions of the cervix and not much on invasive carcinomas. The few studies published so far on Ki-67 expression in invasive cervical carcinomas have shown inconsistent results on the association of Ki-67 with various clinicopathological prognostic factors.

Aims and objectives

To assess Ki-67 expression in cervical carcinomas and to compare it with various clinicopathological prognostic factors.

Materials and methods

Fifty cases of invasive squamous cell carcinoma (SCC) were included in the study. Histological patterns and grades were identified and noted in these cases after microscopic examination of the histological sections. Immunohistochemical (IHC) staining with anti-Ki-67 was done and scored from 1+ to 3+. This score was compared with clinicopathological prognostic factors like clinical stage, histological pattern, and grade.

Result

Among the 50 cases of SCC, 41 showed keratinizing pattern (82%) and nine showed non-keratinizing pattern (18%). Four were in stage I, 25 were in stage II, and 21 were in stage III. Overall, 34 (68%) cases had Ki-67 score 3+, 11 (22%) had Ki-67 score 2+, and five (10%) had Ki-67 score 1+. Ki-67 score of 3+ was the most common score in keratinizing SCC (75.6%), poorly differentiated carcinomas (76.2%), and stage III cases (81%).

Conclusion

We observed statistically significant correlation of Ki-67 expression with higher clinical stage, keratinizing tumours, and poorly differentiated tumours (p<0.05) indirectly implying the poor prognostic significance of this marker.

## Introduction

Carcinoma cervix is the fourth most common malignancy in women worldwide and the second most common malignancy in women in India [1]. Hence, it is crucial to recognize patients with a bad prognosis and initiate the management of such patients early. Even though Papanicolaou (Pap) testing has reduced the number of deaths due to carcinoma cervix, it is still a matter of concern in developing and underdeveloped countries where most of the patients belong to the lower socioeconomic class and resources are limited. Deaths in these patients are usually due to invasion and metastasis due to the aggressive nature of the tumour [2]. Hence, there is a need for new prognostic markers that are easily available and cost-effective to detect high-risk patients with poor prognoses early and treat them early.

Ki-67 is a nuclear antigen associated with cell proliferation. It is seen in all phases of the cell cycle except for the G0 phase. Hence, it is not present in the resting cells. Only cells that over-express p53 or p21 may be evaluated by using Ki-67 because it is present only in dividing cells [3,4]. Normal cervical epithelium shows Ki-67 expression only in parabasal and basal layers [5]. Many studies have been done on the expression of Ki-67 in premalignant lesions of the cervix and its utility as a diagnostic tool [6-8]. But only a few studies have been on Ki-67 expression in invasive cervical carcinomas, which have shown varying results [9,10]. 

This study was done to assess the Ki-67 expression in cervical squamous cell carcinomas (SCC) and to further correlate them with classical known poor prognostic markers like clinical staging, histological grade, and histological pattern to identify their prognostic significance.

## Materials and methods

The study retrospectively included all cases of invasive cervical carcinomas received during the period of June 2015 to June 2018 in the Department of Pathology, SRM Medical College Hospital and Research Centre, Chennai, India. The cases with insufficient tissue material for immunohistochemical (IHC) and cases for which slides or blocks were not available were excluded from our study. After taking into consideration these criteria, 50 cases of cervical SCC were included in the study. Clinical details were obtained from the case sheets and reports from Medical Records Department. In all these cases, the clinical staging was done based on clinical and radiological findings according to the International Federation of Gynecology and Obstetrics (FIGO) staging of cancer of the cervix uteri (2018) [11]. Paraffin blocks and slides of these cases were retrieved from the department archive. Tissue sections of 4-micron thickness were made using a rotary microtome (Leica RM2125; Leica Biosystems, Wetzlar, Germany). These sections were further stained with haematoxylin and eosin (H&E) and evaluated under a light microscope.

In all these cases, the histological pattern was identified and noted based on the 2020 WHO classification of tumours of the uterine cervix [12]. Tumours were also graded into well differentiated, moderately differentiated, and poorly differentiated. IHC staining with anti-Ki-67 (Rabbit monoclonal; PathnSitu Biotechnologies, Secunderabad, Telangana, India) was done in all invasive cervical carcinomas and they were graded as score 1+, 2+, and 3+ based on the following criteria: (i) Score 1+ (Low proliferation): 10-30% positive cells, (ii) Score 2+ (Moderate proliferation): 30-50% positive cells, and (iii) Score 3 + (High proliferation): >50% positive cells

Statistical analysis was done to study the association of Ki-67 with clinical stage, histological grade, and pattern. This was done with IBM SPSS Statistics for Windows, Version 23.0 (Released 2015; IBM Corp., Armonk, New York, United States) using Chi-square test. p-value less than 0.05 was considered statistically significant.

The study was conducted after approval from the Institutional Ethical Committee of SRM Medical College Hospital and Research Centre, Chennai, India (Approval number: IEC 2420).

## Results

In this study, out of 50 cases, 38 (76%) were biopsy specimens and 12 (24%) were hysterectomy specimens. The mean age of patients was 55.4 years with the highest incidence in the age group of 51-60 years. The majority of the patients had complaints of abnormal bleeding per vaginum, which included post-menopausal bleeding (n=23, 46%), heavy menstrual bleeding (n=10, 20%), and post-coital bleeding (n=6, 12%).

After studying the histological findings, it was observed that 41 cases showed keratinizing pattern (82%) and nine showed non-keratinizing pattern (18%). Histological grading was also done in all these cases. Out of the 50 cases, four were well differentiated, 25 were moderately differentiated, and 21 were poorly differentiated. Thus, moderately differentiated SCC was the most common grade in our study. The clinical stage, histological pattern, and grade distribution in biopsy specimens and hysterectomy specimens are given in Tables 1, 2.

**Table 1:** The clinical stage, histological pattern, and grade distribution in biopsy specimens (n=38) SCC: squamous cell carcinoma

Characteristics	Numbers
Clinical Stage	
Stage I	2
Stage II	15
Stage III	21
Histological Pattern	
Keratinizing SCC	32
Non-keratinizing SCC	6
Histological Grade	
Well differentiated	3
Moderately differentiated	19
Poorly differentiated	16

**Table 2:** The clinical stage, histological pattern, and grade distribution in hysterectomy specimens (n=12) SCC: squamous cell carcinoma

Characteristics	Numbers
Clinical Stage	
Stage I	2
Stage II	10
Histological Pattern	
Keratinizing SCC	9
Non-keratinizing SCC	3
Histological Grade	
Well differentiated	1
Moderately differentiated	6
Poorly differentiated	5

**Figure 1:**
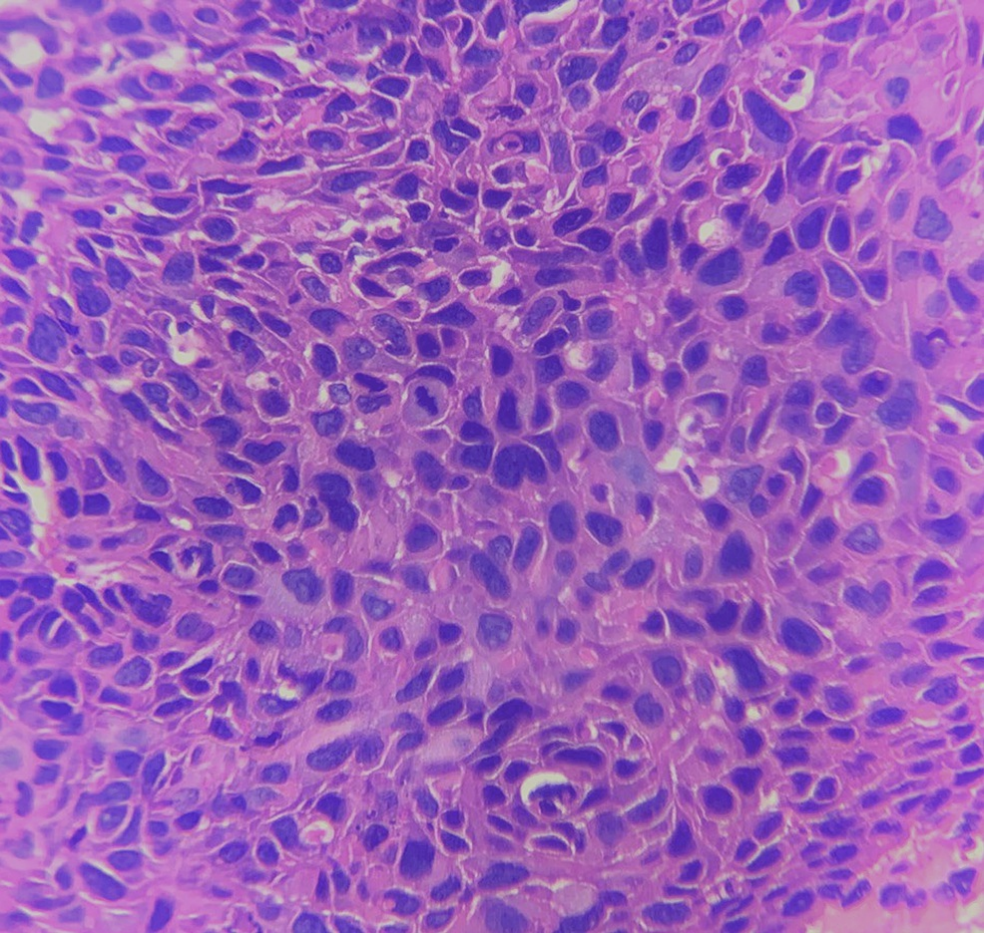
Poorly differentiated squamous cell carcinoma; H&E, X400: neoplastic cells with marked pleomorphism, high nuclear-cytoplasmic ratio, and increased mitosis H&E: hematoxylin and eosin

**Figure 2:**
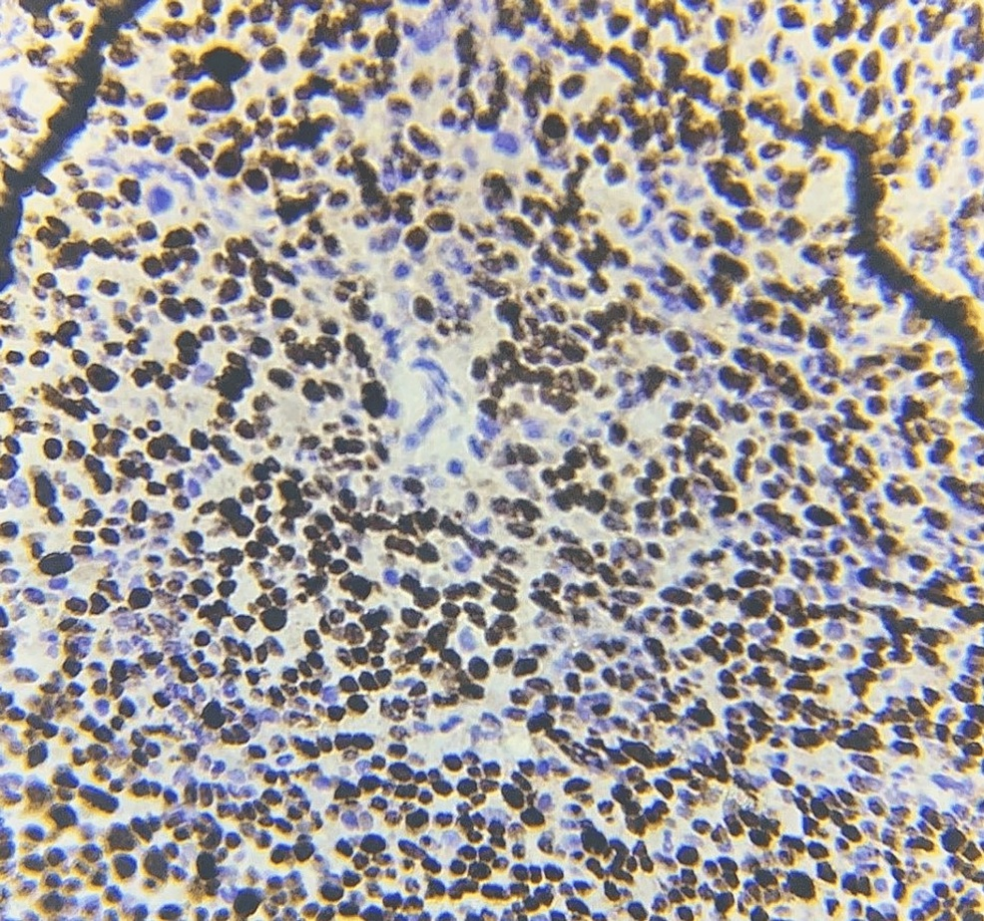
Ki67 score 3+, IHC, X400: more than 50% of cells show nuclear staining pattern IHC: immunohistochemistry

Interpretation of the Ki-67 IHC staining revealed that 3+ was the most common Ki-67 score noted in 34 (68%) cases. Ki-67 score 2+ was noted in 11 (22%) cases and score 1+ in five (10%) cases. Clinical staging by the FIGO system showed that four patients were in stage I, 25 were in stage II, and 21 were in stage III. Thus, stage II was the most common clinical stage in our study. Figure 1 shows an image of poorly differentiated carcinoma and Figure 2 shows an image of Ki67 score 3+.

Association between Ki-67 expression and clinical staging

Among the stage III cases, 3+ was the most common Ki-67 score (81%). None of the stage III cases showed Ki-67 score of 1+. On the contrary, stage I tumours showed predominantly 1+ Ki-67 score (50%). This association between Ki-67 score and the clinical stage was found to be statistically significant (p-0.034) (Table 3).

**Table 3:** Association between Ki-67 expression and clinical staging

Clinical staging	Ki-67 score			Total (n=50)	p-value
	1+	2+	3+		
I	2 (50%)	1 (25%)	1 (25%)	4	0.034
II	3 (12%)	6 (24%)	16 (64%)	25	
III	0	4 (19%)	17 (81%)	21	

Association between Ki-67 expression and histological patterns of SCC

Among the keratinizing SCC, 3+ was the most common Ki-67 score, which was observed in 75.6% of cases, whereas only 33.3% showed Ki-67 score 3+ in non-keratinizing SCC. This association between Ki-67 score and the histological pattern of squamous cell carcinoma was statistically significant (p-0.047) (Table 4).

**Table 4:** Association between Ki-67 expression and histological patterns of squamous cell carcinoma

Histological pattern	Ki-67 score			Total (n=50)	p-value
	1+	2+	3+		
Keratinizing	3 (7.3%)	7(17.1%)	31 (75.6%)	41	0.047
Non-keratinizing	2 (22.2%)	4 (44.4%)	3 (33.3%)	9	

Association between Ki-67 expression and histological grades of SCC

Among the poorly differentiated carcinomas, Ki-67 score 3+ (76.2%) was more frequent than score 1+ (4.8%). However, none of the well-differentiated SCC showed Ki-67 score of 3+. It was also noted that Ki-67 score 1+ was most frequently noted in well-differentiated (50%) SCC than in the other grades. This association between Ki-67 score and histological grades of SCC was also statistically significant (p-0.02) (Table 5).

**Table 5:** Association between Ki-67 expression and histological grades of squamous cell carcinoma

Histological Grade	Ki-67 score			Total (n=50)	p-value
	1+	2+	3+		
Well Differentiated	2(50%)	2(50%)	0	4	0.02
Moderately Differentiated	2(8%)	5(20%)	18(72%)	25	
Poorly Differentiated	1(4.8%)	4(19%)	16(76.2%)	21	

## Discussion

Cervical carcinoma is one of the most common cancers affecting women worldwide, particularly the developing nations. Several studies had shown the increasing incidence of cervical carcinomas in younger ages [13,14]. The survival rate of localised cervical carcinomas (stage IB1) is 91.6% [15]. Thus, it is important to find an easily available prognostic marker for invasive cervical carcinomas.

Ki-67 is a cell proliferation marker which has been extensively studied in intraepithelial lesions of the cervix. But studies showed varied results in Ki67 expression on invasive cervical neoplasms. Since carcinogenesis involves cell proliferation, in this study, we demonstrated the association between Ki-67 and known poor clinicopathological parameters like the clinical stage, histological pattern, and grade of invasive cervical carcinomas.

In the current study, 34 cases (68%) had a Ki-67 score of 3+. In a study done by Gogoi et al., higher expression of Ki-67 (3+) was found in most carcinoma cases (76.32%) [8]. Zhong et al. studied 22 cases of SCC of the cervix, out of which 19 cases showed high-grade expression of Ki-67 [16]. All these findings were similar to this present study with a Ki-67 score of 3+ being predominant in cervical carcinomas.

Clinical stage is a known poor prognostic factor in invasive cervical carcinomas. Wright et al. observed a worse five-year survival rate with higher clinical stages compared to the lower clinical stage [15]. Chen et al. also noted a significantly worse progression-free survival in stage II, stage III, and stage IV tumours compared to stage I [17]. In our study, among the stage III cases, a high Ki-67 score of 3+ was seen in 81% of cases, whereas the stage I tumours had a predominant Ki-67 score of 1+ in 50% of cases. This association between different grades of Ki-67 expression and clinical staging was statistically significant (p-0.034). Similarly, studies by Wu et al. [18], Elsokary et al. [19], Bahnassy et al. [20], and Zhang et al. [21] in cervical carcinomas showed a statistically significant correlation between high tumour stage and high Ki-67 expression. However, Raju et al. [22] and Ancuta et al. [10] did not find any significant association between them. In our study, high Ki-67 was seen more frequently with a high clinical stage of the disease, confirming the significant association between these two parameters.

The histological pattern of cervical carcinoma, particularly keratinizing subtype, is one of the poor prognostic factors. Kumar et al. studied 3,102 cases of keratinizing SCC and 3,751 cases of non-keratinizing SCC and found that keratinizing SCC was more likely to be in the advanced stage and were independent predictors of survival [23]. Randall et al. also observed lower survival rates of 40% in keratinizing SCC compared to non-keratinizing large cell carcinoma (61%) [24]. In the current study, keratinizing SCC (75.6%) showed a more frequent high Ki-67 expression score 3+ than the non-keratinizing SCC (33.3%). This was statistically significant (p = 0.047). Only a few studies demonstrated the association between Ki-67 expression and histological patterns. Carrilho et al. [25] and Mishra et al. [26] reported that high Ki-67 was associated with the keratinizing histological pattern. Thus, this study confirmed the suggestion from these two previous studies that keratinizing histological pattern was significantly associated with high Ki-67 expression.

The histological grade is also found to be an independent prognostic factor for disease-free survival and overall survival in other studies [27,28]. Brambs et al. noted a significant reduction in recurrence-free survival in patients with poorly differentiated tumours [28]. Matsuo et al. observed an association between higher-grade tumours and decreased survival [29]. In this study, among the poorly differentiated carcinomas, the Ki-67 score of 3+ (76.2%) was more frequent than the score 1+ (4.8%). It was also noted that the proportion of Ki-67 score 1+ was higher in well-differentiated tumours (50%) than in poorly-differentiated tumours (4.8%). This association between Ki-67 expression and histological grade was statistically significant (p = 0.02). Few authors had previously observed a significant association between Ki-67 expression and histological grade [8,30]. But Zhang et al. did not observe a significant association between these two parameters [21]. Our study results confirmed the former findings that there is a significant association between higher Ki-67 expression and histological grades of invasive cervical carcinomas.

In this study, we demonstrated the association between high Ki-67 expression and known adverse clinicopathological parameters in invasive cervical carcinomas, suggesting the prognostic significance of Ki-67 in invasive cervical lesions. However, it is worth noting that there are some limitations regarding our study. We had 50 cases of invasive SCC in our study. Particularly the number of well-differentiated SCC and stage I tumours was relatively less. Future studies with more representation of stage I tumours and well-differentiated SCC will help in confirming our results. Follow-up data was also not available for many of these patients. Hence, we were not able to study the impact of various degrees of Ki-67 expression on the survival of patients with cervical SCC. More studies with follow-up and survival analysis are needed to study the impact of Ki-67 expression on the survival of patients with cervical SCC. 

## Conclusions

We noted a statistically significant correlation of Ki-67 expression with higher clinical stage, keratinizing tumours, and poorly differentiated tumours. This indicates that Ki-67 is an adverse prognostic marker in invasive cervical carcinomas. However, this needs to be confirmed by larger-scale studies on the impact of various degrees of Ki-67 expression on patient survival. After such confirmation, Ki-67 can also be used as a routine prognostic marker for cervical carcinomas, as we do for breast carcinomas. This may help in identifying the patients with poor prognosis and for such patients, more aggressive treatment or intense follow-up could be recommended.
